# Left Main Coronary Artery Fusiform Aneurysm

**DOI:** 10.7759/cureus.24790

**Published:** 2022-05-06

**Authors:** Miguel A Rodriguez Guerra, Ana P Urena Neme, Michael Victoria, Gabriella Roa Gomez, Giancarlo Acosta

**Affiliations:** 1 Medicine, Montefiore Medical Center, Albert Einstein College of Medicine, Bronx, USA; 2 Cardiology, Medicina Cardiovascular Asociada, Santo Domingo, DOM; 3 Medicine, Instituto Tecnologico de Santo Domingo, Santo Domingo, DOM; 4 Pulmonary and Critical Care Medicine, Montefiore Medical Center, Albert Einstein College of Medicine, Bronx, USA; 5 Cardiology, Marshall University, Huntington, USA

**Keywords:** polyarteritis nodosom, vasculitis, fusiform coronary aneurysm, left main coronary artery aneurysm, coronary aneurysm

## Abstract

Aneurysm of the coronary artery is an uncommon condition that is usually found incidentally. The left coronary aneurysm is the least common. We report the case of a young patient with a history of vasculitis who was found to have a left fusiform coronary aneurysm. This is a 20-year-old female who has a history of polyarteritis nodosa and who came due to shortness of breath associated with chest discomfort. The physical exam was only relevant for multiple joint pains and tenderness. An echocardiogram showed a possible coronary aneurysm that was confirmed on the angio-tomography. The patient was discharged without complications. The left main coronary artery aneurysm is a rare condition and the least common of the coronary aneurysms. There is no established guideline for screening and therapy of these aneurysms, but invasive methods are not a preferred method for follow-up on this condition.

## Introduction

Aneurysms of the coronary arteries (CAA) are uncommon, incidentally found, and usually asymptomatic [1,2]. The left CAA is the least common. Multiple conditions have been associated with aneurysms, including syphilis, vasculitis, autoimmune diseases, and congenital [3,4]. The coronary arteries could be studied with multiple techniques, but CAA is ideally monitored with non-invasive methods like echocardiogram (ECG), computed tomography angiography (CTA), and magnetic resonance angiography (MRA) of the coronaries imaging [5,6]. This is a case of a young patient with vasculitis who was found to have a fusiform aneurysm of the left main coronary artery that extended to the left coronary bifurcation without major complications.

## Case presentation

**Figure 1:**
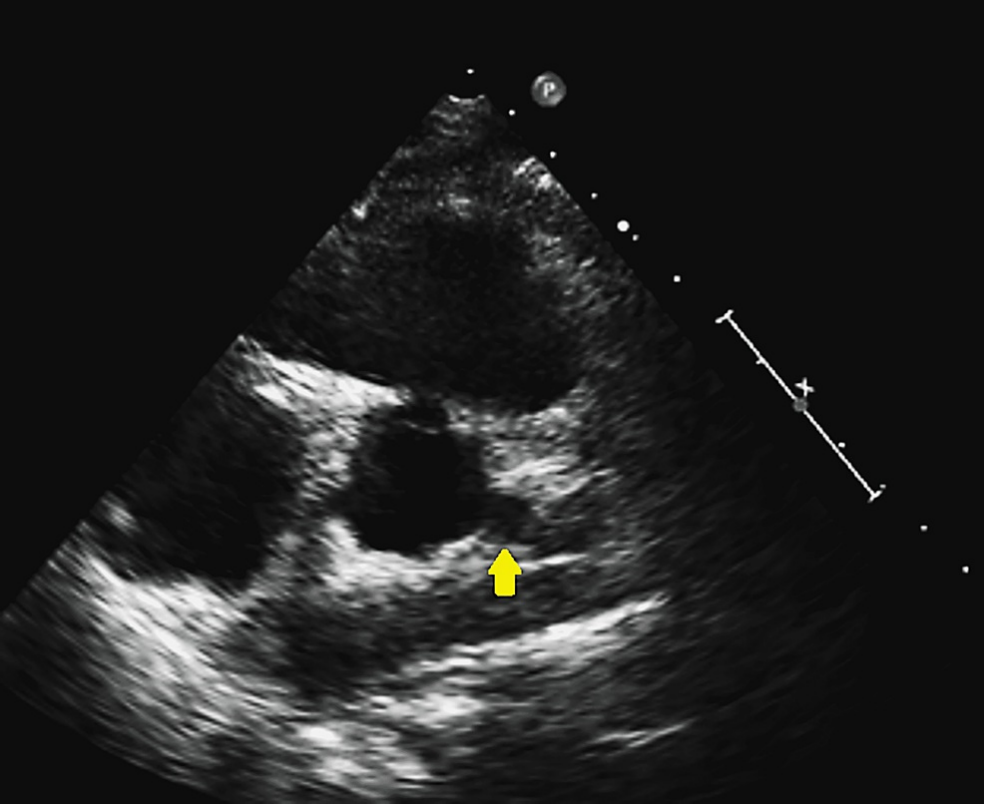
Echocardiogram showing a possible left main aneurysm. Yellow arrow indicated the possible left main aneurism.

The patient was a 20-year-old African-American female with a history of polyarteritis nodosa, complicated with uveitis and leukocytoclastic vasculitis, who presented due to persistent shortness of breath that interferes with her regular activities and is associated with chest discomfort. She was recently started on anticoagulation after a lupus anticoagulant test resulted in a positive. She is also on Depo-Provera for contraception; an intrauterine device was recommended but she did not accept it. The physical examination showed a young female without distress with multiple joint pains and tenderness. Her ECG showed normal sinus rhythm, but due to her vasculitis history, an echocardiogram was ordered and showed a possible left main fusiform (Figure 1).

**Figure 2:**
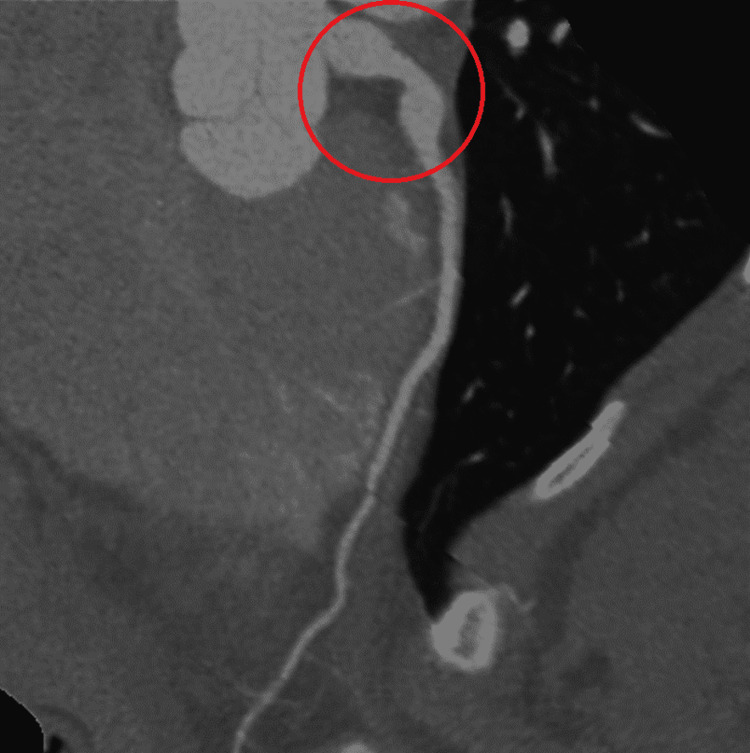
CTA coronaries show the aortic root and the left coronary aneurysm. Red circle indicates the left main fusiform aneurysm. CTA: computed tomography angiography.

**Figure 3:**
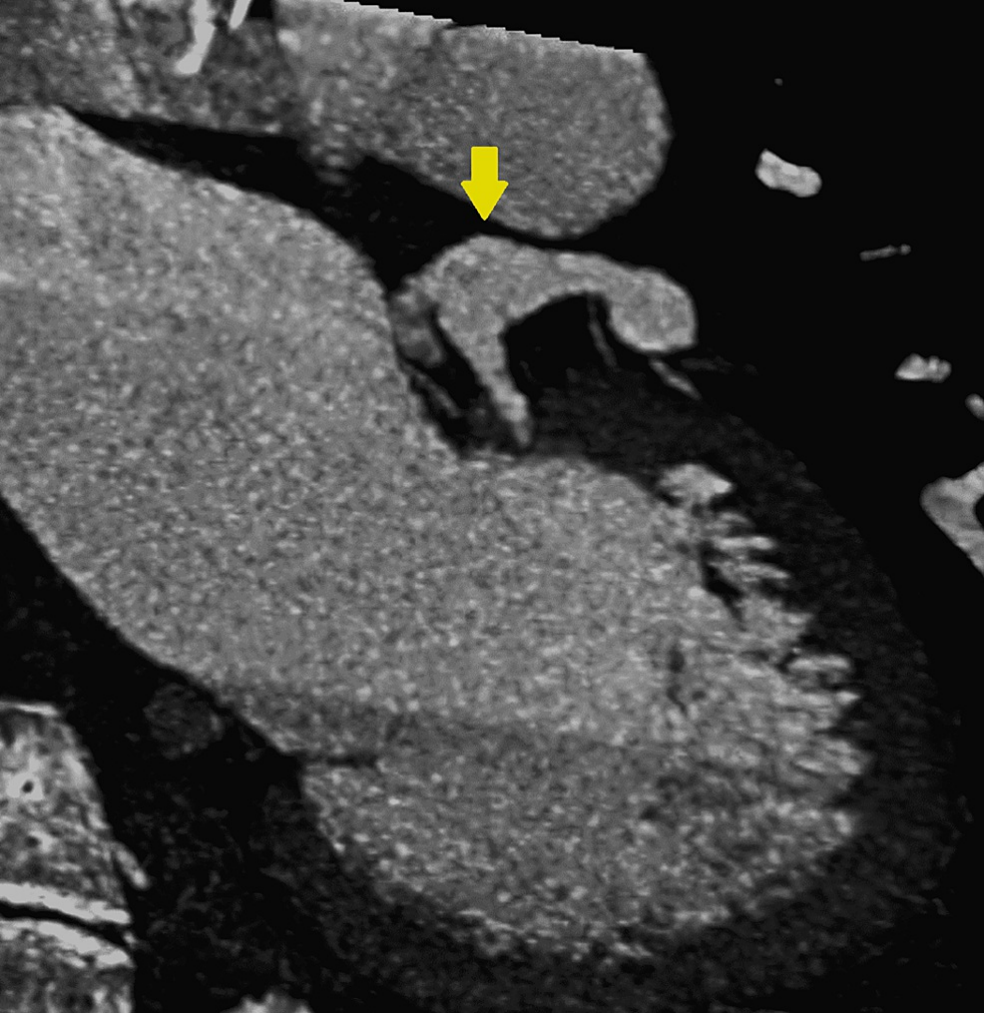
CTA coronaries showing the ventriculogram and the left coronary fusiform aneurysm. Yellow arrow indicates the fusiform aneurysm. CTA: computed tomography angiography.

A computed tomography angiography (CTA) re-demonstrated the left coronary aneurysm measuring 10 mm (Figure 2, 3).

The patient and her family opted for preventive anticoagulation. A post-discharge exercise stress test was done and showed augmentation of contraction in all visualized segments during the peak exercise, and it was negative for ischemia-induced wall motion abnormality.

## Discussion

The aneurysm of the coronary arteries (CAA) is defined as a localized dilation of a coronary segment that is at least one time and a half of the regular arterial size. Their incidence stands between 0.3% and 5.3%, is usually caused by coronary artery disease (CAD), and is more common in males (2.2% vs. 0.5%) [7,8]. Besides CAD, there are other conditions related to CAA, vasculitis, connective tissue disorders, and intravascular lesions, including complications of drug-eluting stents [9].

The CAA is usually asymptomatic and therefore, discovered incidentally. In addition, their presence is associated with poor long-term prognosis independently of coronary artery disease. Clinical presentations vary depending on the cause of the aneurysm; in atherosclerotic disease, the most common symptom is angina pectoris [8,9].

According to some data, CAA has been incidentally found during coronary angiography in 0.9% to 4.9% of patients, and its presence has been related to a worse clinical outcome. Morphologically, there are also two main types of aneurysms: saccular, which is defined as a transverse dilation greater than the longitudinal diameter, and fusiform, which has a bigger longitudinal diameter.

The right coronary artery is the most affected by aneurysms, seen in almost 40%-70% of patients, followed by the left anterior descending artery in 32.3% of the cases and then the left circumflex in 23.4% [10]. Multivessel involvement or left main coronary aneurysms are less frequent and only seen in about 3.5% of the population [1].

The left main CAA is a rare entity generally associated with atherosclerosis [11]. The most extensive study of left main aneurysms was performed by Doustkami et al. Out of 20,332 patients, only 22 cases (0.1%) were reported. In Syed's study, 3,200 coronary angiograms were reviewed, and only 3% of the aneurysms were found in the left main [12].

Ostial coronary aneurysms are described most frequently in patients with connective tissue disorders. In Harikrishnan's study, ostial lesions were found in 58.1% of Marfan patients that had previously undergone aortic root replacement [13]. In addition, Haroun et al. reported a 43% prevalence of ostial coronary aneurysms in Marfan patients, especially those under 35 years old [14].

In terms of complications, the CAA tends to be a silent entity discovered incidentally, but it may have fatal complications; the flow stasis and reduction of shear stress result in thrombosis and embolization. In the presence of obstructive atherosclerotic disease, CAA can cause angina due to aneurysmal artery spasms or acute coronary syndrome (ACS); they could also predispose to sudden death, and cardiac tamponade can ensue if they rupture [9]. Although uncommon, an aneurysm could undergo thrombotic changes leading to acute myocardial infarction (AMI), but the incidence of AMI in CAA remains unknown. Forte et al. reported that only 22.3% presented with atypical chest pain out of the 66.7% with intraluminal thrombosis. Furthermore, arrhythmias can occur if the enlargement compresses adjacent structures such as the right atria (RA), right ventricle (RV), pulmonary artery, or sinus node (SN) [15]. As a result, the treatment should be individualized depending on the clinical presentation, type of aneurysm, size, location, patient demographics, and the physician's experience.

The treatment options for CAAs consist of surgical, percutaneous coronary intervention (PCI) (stent angioplasty and coil embolization), and medical approaches. Unfortunately, there are no established guidelines or standardized recommendations for managing coronary artery aneurysms, but the risk factors' control and lifestyle modification need to be added regardless of the presence of obstructive coronary artery disease [16].

The use of statins is recommended due to their pleiotropic effect, but the antiplatelet or anticoagulation therapy is controversial, and its use must be based on the risk for thrombosis. Historically percutaneous coronary intervention (PCI) is preferred in smaller aneurysms, and surgical intervention is preferred for larger ones when intervention is deemed necessary [16].

Multiple surgical techniques have been described. They include aneurysmectomy with or without coronary artery bypass graft and aneurysm ligation or resection, but a guideline of which one is the best procedure has not been established [17]. Surgery is an alternative for symptomatic patients with concomitant CAD, CAA on the left main, or giant CAA, and before deciding on excision, short and long-term risk needs to be considered. Covered stents appear to be the best choice if the percutaneous exclusion is suitable based on anatomy [18].

When the CAA is the culprit vessel in coronary syndrome, the goal is to restore the flow. The treatment modality will be decided based on the aneurysm's shape and extension in high-risk lesions or patients [19]. A covered stent could be used on aneurysms that do not involve a significant side branch. In contrast, when it involves a significant side branch, a balloon or stent-assisted coil embolization would be required, or surgical intervention will be required. If the CAA involves the left main coronary artery and multiple or giant CAAs, surgery is considered first; and in cases with symptomatic external compression, percutaneous intervention with occluder devices or coil embolization would be the alternative [20].

## Conclusions

There are no guidelines to screen for coronary aneurysms, even in the presence of vasculitis; their diagnosis is mostly an incidental finding. The left main coronary artery aneurysm is a rare condition and the least common of the coronary aneurysms; the literature has associated it with vasculitis. The best method to study this condition would be coronary angiography, but a non-invasive method is preferred. This condition has to be monitored but does not necessarily represent a major risk for the acute coronary syndrome.
